# Norfloxacin Loaded pH Triggered Nanoparticulate *in-situ *Gel for Extraocular Bacterial Infections: Optimization, Ocular Irritancy and Corneal Toxicity

**Published:** 2016

**Authors:** Preeti Upadhayay, Manish Kumar, Kamla Pathak

**Affiliations:** *Department of Pharmaceutics, Rajiv Academy for Pharmacy, Chattikkara, Mathura, India.*

**Keywords:** Norfloxacin, Nanoparticulate *in-situ* gel, Factorial design, Ocular irritancy, Corneal toxicity

## Abstract

In order to achieve prolong corneal contact time of norfloxacin (NFX) for treatment of extra ocular diseases, a pH triggered nanoparticulate *in-situ* gelling system was designed to explore dual advantage of nanoparticles and *in-situ* gelling system, for its ocular delivery. NFX loaded nanocarriers were developed by ionotropic gelation technique using chitosan as a matrix forming polymer, cross-linked by an anionic crosslinker sodium tripolyphosphate (TPP). Optimization of nanoformulations was done by 3^2 ^full factorial design using chitosan and TPP concentration(s) as the independent variables and particle size, % entrapment efficiency and % cumulative drug release as the responses. The experimental design was validated by extra design check point formulation (N10). The optimized formulation (N4) selected on the basis of highest desirability factor (0.895) was developed as *in-situ* gelling system using carbapol934 and evaluated. The best in-situ gelling formulation (N4G5) was sufficiently mucoadhesive, corneal toxicity, antibacterial activity and free from ocular irritancy.

## Introduction

Extraocular bacterial infections require an antibiotic treatment regimen to reduce the complications associated with chronic conditions (1). The success of an antibacterial therapy depends upon the achieving and maintaining the therapeutic concentration of drug at the site of infection for a prolonged duration (2) without compromising the intraocular region (3). Many antibacterial agents are available for treatment of bacterial infections and norflaxacin (NFX) is widely used in treating the extraocular infections. It is a broad spectrum fluoroquinolone antibiotic which potentially acts by inhibition of bacterial enzymes DNA gyrase and topoisomerase IV, responsible for bacterial cell division (4). Marketed as eye drops, rapid precorneal drainage requires frequent instillation or use of high drug concentration which usually leads to a pulse kinetics pattern of drug concentration (5). 

To conquer these problems many novel delivery systems have been researched that include ocuserts (6), *in-situ* gelling system (7), imprinted soft contact lenses (2), microspheres (8) and liposomes (5). These novel systems are not free from limitations that can be overcome by nanocarrier systems, mainly because the association of an active agent to a nanocarrier allows the drug molecules to interact with the specific ocular structures and thus override the ocular barrier and prolong the residence time at target area (9). Nanoparticles due to their small size are efficacious delivery systems, have longer retentivity in the inflamed region as compared to the normal eyes, indicating their potential to target inflamed tissue (10). Generally biodegradable nanoparticles of natural polysaccharides have attracted the interest of researchers due to their desirable properties such biocompatibility, biodegradability and protective actions (11-13). Chitosan is a biodegradable polysaccharide derived by partial deacetylation of chitin and is widely used for ocular delivery. Other typical activities of chitosan include antitumour activity, acceleration of wound healing, and antibacterial activity which have lead to its suitability as drug carrier systems (14). Ionic crosslinking of chitosan with crosslinking between the positively charged primary amino group of chitosan with pentavalent polyphosphate anion can yield nanosized colloidal particles However, the residence time of colloidal system is comparatively low in the ocular region and to enhance it, viscosity modulation can be attempted. 

*In-situ* gelling systems are advantageous as these are based on the concept of phase transition in response to the environmental changes. Such systems offer the advantage of ease of administration as sol form that converts to gel at the site of application ensuring enhanced retention (15). Thus the aim of study was to combine the advantages of the nanoparticles and *in-situ* gel to obtain a NFX formulation with improved residence at the site of application along with the ability to provide a sustained release. The study was performed in two stages; first step included preparation of nanoparticles by ionotropic gelation technique which is nontoxic, organic solvent free, convenient and controllable method of nanoparticles preparation. The NFX loaded nanoparticles were optimized by 3^2^ full factorial design and characterized for physiochemical properties, morphology and release characteristics. The second stage involved preparation of nanoparticulate *in-situ* gel that was evaluated for release characteristics, rheology, corneal toxicity and ocular irritancy. 

## Experimental

*Materials*

Chitosan with 75-85% deacetylation of medium viscous grade, viscosity 200.20 cps (1% solution in 1% acetic acid) were purchased from Sigma Aldrich, New Delhi. Sodium tripolyphosphate (TPP) was purchased from Balaji chemicals, Vadodara, Gujarat. Norfloxacin was obtained as a gift sample from Intas Pharmaceutical, Dehradun. Carbopol 934P was obtained from. Himedia Central Drug House, New Delhi. Dialysis membrane 150 was procured from Qualigens Fine chemicals, Mumbai, India. Acetic acid was purchased from S.D. Fine chemicals, Mumbai, India. 

*Preliminary trials*

Preliminary trials were performed in order to determine the zone of nanoparticles formation to select the concentration of chitosan and TPP to be used in the experimental design. The nanoparticles were prepared by ionotropic gelation technique developed by Calvo et al. (16). Briefly, chitosan was dissolved in 0.2% v/v acetic acid while TPP was dissolved in water. The TPP aqueous solution was added dropwise to chitosan solution under constant magnetic stirring at high speed. The developed formulations were physically examined for clear solution, opalescent suspension and aggregates that settled subsequently. The zone of nanoparticles formulation was demarcated. 

*Preparation of NFX loaded nanoparticles *

The levels of chitosan and TPP were selected and NFX loaded nanoparticles were made by incorporation technique using the experimental design (Table 1). In the above detailed method, NFX was dissolved in chitosan solution to obtain a clear solution and the pH was adjusted to 4.8 using 1 N NaOH to ensure complete protonation of chitosan. Finally aqueous solution of TPP was added dropwise to the drug-chitosan solution under constant magnetic stirring. The mixture was left stirring for 1 hour to ensure maximum drug loading. The nanoparticles were collected by centrifugation (REMI high speed, cooling centrifuge Remi Corporation, India) at 10,000 rpm for 15 min at 4 °C and redispersed in 10 mL of deionized water.

**Table 1:** Full factorial experimental design layout

**Formulation code **	**NFX** **(% w/v)**	**X**_1_	**X**_2_	**Dependent variables**
		**Chitosan %(w/v)**	**TPP** **%(w/v)**	
N1	0.1	0.1 (-1)	0.05 (-1)	
N2	0.1	0.1(-1)	0.075 (0)	
N3	0.1	0.1 (-1)	0.1 (+1)	Y1= % cumulative drug release
N4	0.1	0.15 (0)	0.05 (-1)	Y2= % entrapment efficiency
N5	0.1	0.15(0)	0.075 (0)	Y3= % entrapment efficiency
N6	0.1	0.15(0)	0.1(+1)	
N7	0.1	0.2 (+1)	0.05 (-1)	
N8	0.1	0.2 (+1)	0.075(0)	
N9	0.1	0.2 (+1)	0.1 (+1)	
N10*	0.1	0.125 (-0.5)	0.63 (-0.5)	

* Extra design check point formulation

**Experimental **

The effect of independent variables, chitosan and TPP concentration, set at three levels were studied on the response variables namely, particle size, % entrapment efficiency and % cumulative drug release. Mathematically the results were expressed as second order polynomial equation-

Equation 1 $$Y_{i}=b_{0}+b_{1}X_{1}+b_{2}X_{2}+b_{12}X_{1}X_{2}+b_{11}X_{1}^{2}+b_{22}X_{2}^{2}$$

Where b_i_ is the estimated coefficient for factor X_i_ and Y_i_ which are the measured response coefficients corresponding to linear effects b_1_ and b_2_, interaction effects b_12_ and quadratic effects b_11_ and b_22_. An extra design checkpoint (N10) was constructed for validating the experimental design. Statistically the polynomials obtained were validated by using the ANOVA provisions of design expert software. To study the combined effect of different variables on the responses, 3-D response surface plots were generated. 

*Characterization of nanoparticles*

*Particle size and zeta potential*

The mean particle size, zeta potential and polydispersity index of NFX loaded nanoparticles were determined using photon correlation spectroscopy with Malvern Zetasizer (Malvern instruments ltd, UK). The size analysis was performed at an angle of 90 °C based on light scattering phenomena using samples diluted with water. The hydrodynamic diameter was calculated from autocorrelation function of the intensity of light scattered from particles with the assumption that the particles have a spherical form. Sample volume used for analysis was kept constant i.e. to nullify the effect of stray radiation from sample to sample.

*Entrapment efficiency*

The entrapment efficiency of nanoparticles was determined by an indirect method in which NFX loaded nanoparticles were separated from the aqueous medium containing non-associated NFX by ultracentrifugation in cooling centrifuge (Model no. C-24 BL, REMI corporation, India) at 10,000 rpm (7826 g) for 15 min. The amount of free drug in the supernatant was determined spectrophotometrically at 272 nm. The entrapment efficiency of nanoparticles of nanoparticles was determined in triplicate and calculated as follows.

Equation 2 $$\mathrm{Entrapment efficiency}=\frac{\mathrm{Total amount of drug}-\mathrm{Unentrapped drug}}{\mathrm{Total amount of drug}}\times 100$$

*In-vitro* *release*

The *in-vitro* release studies were conducted by dialysis bag technique. After separation of un-entrapped drug and washing procedure by centrifugation, nanoparticulate pellet was dispersed in small volume of simulated tear fluid (STF; made with sodium chloride-0.67g, sodium bicarbonate-0.20g, calcium chloride dihydrate -0.008g in distilled water q.s 100 mL) (17,18) and the volume made up to 10 mL. The redispersed nanoparticulate sample equivalent to 3 mg NFX were placed in dialysis bag (cellulose membrane, Mol. wt cut off 12- 14 kD, sigma) and tied at both ends. Dialysis bag was immersed in 50 mL of STF, maintained at 37^ o^C and stirred at 50 rpm. Three milliliter of sample was withdrawn from the receptor compartment during each hour up to 12 h including t = 0 h and replaced with fresh media to maintain sink conditions. The samples were assayed for drug spectrophotometrically. Triplicate runs were performed for each formulation. 

*Selection of optimized formulation*

The optimized formulation was selected on the basis of desirability values obtained upon appli

cation of constraints in the statistical design, which included minimization of particle size, maximization of % entrapment efficiency and % cumulative drug release. The formulation with maximum desirability factor was selected for further studies.

*Diffuse reflectance spectroscopy (DRS)*

DRS spectroscopy was conducted for NFX, chitosan, physical mixture, and N4. The samples were mixed with KBr (IR grade) and analyzed by diffuse reflectance spectrophotometer (Shimadzu- 8400S, Kyoto, Japan) with DRS attachment using potassium bromide spectrum as background and sample spectrum was overlapped on it. The scanning range was 500-4000 cm^-1^. The characteristics peaks were recorded for different samples.

*Nanoparticulate in-situ Gel*

*Formulation*

The* in-situ* gelling system was formulated by dispersing optimized formulation N4 into the polymeric solution of carbopol 934P. The *in-situ* gels were prepared by dispersing nanoparticles equivalent to 30 mg of NFX in 10 mL of carbopol base so that the final concentration of NFX in the *in-situ* gel was 0.3 % w/v. The composition of nanoparticulate *in-situ* gels (N4G1 – N4G5) is described in Table 2.

**Table 2:** Composition of nanoparticulate *in-situ* gel of norfloxacin

**Formulation code**	**NFX** **(% w/v)**	**CS** **(%w/v)**	**TPP** **(% w/v)**	**Carbopol 934P** **(% w/v)**
N4G1	0.3	0.15	0.05	0.1
N4G2	0.3	0.15	0.05	0.2
N4G3	0.3	0.15	0.05	0.3
N4G4	0.3	0.15	0.05	0.4
N4G5	0.3	0.15	0.05	0.5

*Evaluation*

*Clarity and Optical transmittance*

The clarity of developed formulation was determined before and after gelation by visual examination of formulation under light against white and black background. Optical transmittance of *in-situ* gel was determined spectrophotometrically (19). The samples of *in-situ* gel were transferred to quartz cuvette and the transmission of light was measured at 480 nm with STF as reference. 

*pH and Dug Content*

The pH of formulations, N4G1-N4G5 was measured by Digital pH meter; model 111 E (HICON, New Delhi, India). Drug content was determined by diluting 100 mg of the sol to 5 mL with ethanol (95%) and vortexing for 5 min. The volume was made up to 10 mL with phosphate buffer, pH 6.8 and assayed spectrophotometrically. Accurately weighed quantity of gel equivalent to 10 mg of NFX was extracted with 5 mL of 1% v/v acetic acid solution and filtered to discard the residue. The extract was transferred to 100 mL volumetric and volume made up with simulated tear fluid, which was further analysed spectrophotometrically at 272 nm.

*In-vitro gelling*

The nanoparticulate *in-situ* gels were evaluated for their gelling ability in response to the pH changes encountered on ocular administration. The formulations were transferred to a test tube individually and mixed with STF in a ratio of 25:7 to imitate the *in-vivo* ocular conditions (19). The gelling ability was determined by visual inspection and graded according to their *in-situ* gelling time and dissolution time which included: system(s) with slow gelation, immediate gelation and immediate gelation with extended duration of dissolution. Based on the evaluations detailed in preceding sections, best *in-situ* gelling system was selected and was subjected to battery of tests described below.

*Viscosity*

The rheological behaviour of N4G5 (best *in-situ* gel) was evaluated by Brookfield viscometer DV-II^+^ pro (Brookfield Engineering Laboratories, Inc, MA) coupled with T spindle S-96, at 37 ± 2 ˚C. The formulation was placed in 10 mL beaker and the spindle was lowered perpendicularly taking care that it did not touch the bottom of the beaker. The spindle was rotated at 5 rpm and readings were recorded after they became constant. The method was repeated for 10, 20, 50, 80 and 100 rpm. Similarly to assess the viscosity profile at physiological pH the formulation pH was raised to 7.4 using 0.5 M NaOH and the process was repeated as mentioned above (20). 

*In-vitro release*

*In-vitro* release study of NFX nanoparticulate *in-situ* gel was performed by vertical Franz diffusion cell. The release profile was compared against control gel consisting of 0.3% w/v NFX and 0.5 % w/v carbopol 934P. For conducting the study donor compartment of the vertical Franz diffusion cell was filled with 1 mL formulation while the receptor compartment was filled with 18 STF, both the receptor and donor compartment were separated by dialysis membrane (M.Wt cut off 12-14 kD) presoaked in STF overnight. The assembly was maintained at 37 °C with the help of water bath and the receiving fluid was maintained at constant magnetic stirring. 1 mL sample was withdrawn at regular intervals and replaced with fresh media to maintain the sink condition. The sample obtained were analysed spectrophotometrically at 272 nm. Experiments were conducted in triplicate (21). 

*Transmission Electron Microscopy*

Morphological analysis of N4G5 was performed using Transmission electron microscope (FEI Technai G^2^, USA). The sample (5-10 µL) was dropped on carbon coated grids and dried. The dried samples were stained with 2% w/v phosphotungstic acid and images were obtained at an acceleration voltage of 200 KV. The morphology of N4G5 was compared against reference image of N4 nanoparticles.

*In-vitro mucoadhesion*

**Figure 1:**
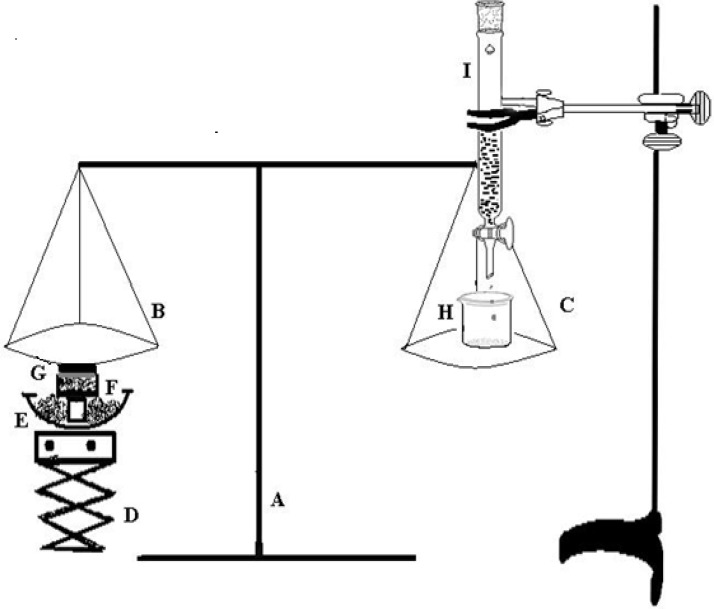
Mucoadhesive force measuring assembly: (A) balance;(B) left pan;(C) right pan;(D) height adjusting pan; (E) water bath; (F) sample holder; (G) corneal tissue; (H) beaker; (I) burette

The mucoadhesive strength of gel was determined by the method reported by Qui et al (22). The experimental setup for determination of mucoadhesive force is shown in Figure 1. The corneal tissue was washed with normal saline, and placed in freshly prepared glutathione bicarbonate Ringer’s solution at 35 °C for 10 min. The corneal tissue (G) was then excised and attached to the undersurface of pan (B) of balance (A). The balance was equilibrated and the optimized *in-situ* gel N4G5 was placed in the sample holder (F) kept in a water bath (E) maintained at 37°C. Finally the pH of the sample was adjusted to 7.4 using 0.1 M NaOH. The equilibrium of balance was destroyed by placing a weight of 5 g on the left pan of balance (B) to assure the contact between the corneal tissue and gel surface. The weight was then removed and the balance was allowed to regain its equilibrium. Thereafter, water was added dropwise to the beaker (H) placed on right pan (C) of balance with the help of a burette (I). Water was continuously added to the beaker until cornea was detached from the gel. The mucoadhesive force was determined as the detachment stress (dynes/cm^2^) which gives the value of minimal force required to detach the cornea from the gel. The experiment was done in triplicate and corneal pieces were changed for each measurement. 

*Corneal toxicity*

To determine the effect of formulation on corneal structure and integrity histological studies were conducted. Excised cornea was washed with normal saline for 1 min and incubated with N4G5, phosphate buffer saline, pH 7.4 (negative control) and 75% v/v isopropyl alcohol (positive control) separately for 30 min. The corneas were thereafter washed with phosphate buffer saline, pH7.4 and immediately fixed with 10% v/v formalin solution for 24 h. Corneas were dehydrated with ethyl alcohol gradient (70-90-100%) and xylene; immersed in melted paraffin and solidified in block. Finally cross sections (<1 mm) were excised and mounted on glass slide and stained with hematoxylin and eosin and observed for histological changes in tissue sections with respect to controls. 

*Anti bacterial activity*

The antimicrobial activity of the formulation was performed to ascertain the activity of *in-situ* gel against the microbial agent (*S. aureus*). The anti bacterial activity was performed according to the cup-plate method as prescribed in I.P. 2007 (23). According to which a layer of nutrient agar media (20 mL) was seeded with the innoculum of test organism (0.2 mL) and allowed to solidify in petriplates. In the solidified media cups were bored with the help of sterile cork borer of 3mm dia. Then a fixed volume i.e. 100 µL of formulation (optimized and marketed) containing equal amount of drug was poured into the cups bored onto the media. Along with preparation of test and standard a negative and positive control were also prepared which consisted of uninoculated media and media seeded with test organism but deprived of antibacterial agent. The prepared petriplates were left at room temperature for 4 h then further incubated at 37 °C for 24 h. The diameter of zone of inhibition was measured at 18 and 24 h with the help of microbial zone reader.

*Ocular irritation potential*

To evaluate the ocular irritation potential of N4G5 formulation HET-CAM test was performed. The chorioallantoic membrane (CAM) was developed in freshly collected fertile eggs. The eggs were incubated at 37 °C ± 0.5 °C and 55 ± 5 % RH for three days. The eggs were candled to check the viability of the eggs and those found to be nonviable were discarded. The viable eggs were further incubated for 10 days with manual rotation of egg at every 12 h to ensure proper growth of CAM. On 10^th^ day 10 the air cell was marked and the shell was cut off and removed. The underlying membrane was moistened carefully with 0.9% NaCl at 37 °C. The moistening solution was carefully poured out from the opened egg and the membrane was carefully removed to ensure that there was no damage to the underlying blood vessels. The exposed CAM was then treated with 0.3 mL of test formulation. Separately, CAM was also treated with 0.3 mL each of 1N NaOH (positive control) and 0.9% w/v NaCl (negative control) and incubated at 37 °C ± 0.5°C and 55 ± 5% RH for three days. The CAM was analyzed for changes, if any, in the vascular structure (hemorrhage, lysis and coagulation) due to the treatments. The changes in CAM were scored according to the scoring chart for HET-CAM test (24).

## Results and Discussion

*Preparation of chitosan nanoparticles*

**Figure 2:**
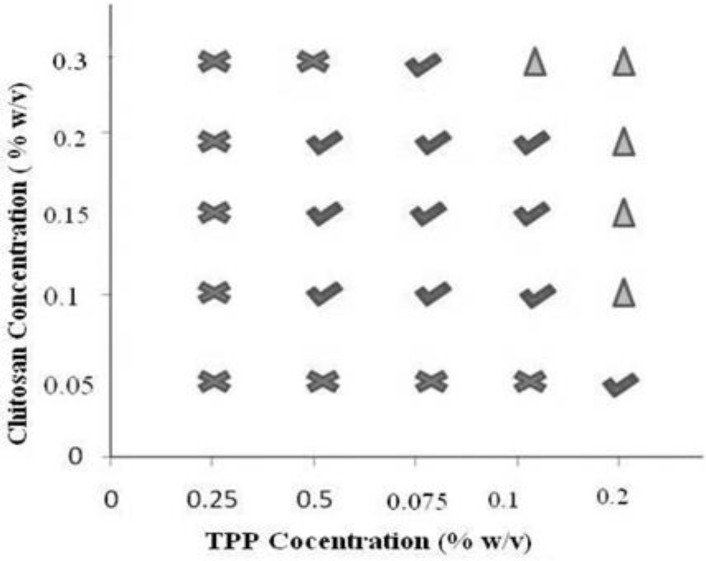
Phase diagram for preliminary trials for determination of chitosan and TPP concentration.

Formulation of chitosan nanoparticles by ionotropic gelation technique requires a strict control over the formulation conditions as a narrow range of concentration is available for the formation of chitosan nanoparticles (24). To ascertain the zone of nanoparticle formation preliminary trials were conducted by varying chitosan and TPP from 0.05 - 0.3 and 0.025 - 0.2 % w/v respectively. Consequently, three types of systems were identified which included clear solution, opalescent suspension, and aggregates depending on the ability of the crosslinker and polymer to interact for particles of nanometric range that in turn depends on the concentration. Polyphosphate anion from TPP cannot crosslink the polymeric chain of chitosan adequately at low concentration i.e. 0.025% hence a clear solution was obtained at low concentration of TPP irrespective of the concentration of chitosan. When the concentration of TPP was raised to 0.2% w/v aggregates were formed due to extensive crosslinking caused by the TPP at every concentration of chitosan. Apart from concentration, pH of chitosan solution also affects nanoparticle formation, as chitosan is a weak polyelectrolyte with pKa around 6.5, its degree of protonation is exclusively controlled by pH. As dictated in literature an increase in pH of chitosan solution from 4.7 to 8 results in decline of degree of protonation from 100 to 0% and hence crosslinking (11). Therefore the pH of reaction mixture was maintained at 4.8 using 0.1 M NaOH to ensure maximal protonation of chitosan. The pH selected can also be considered optimum that can minimize probability of undesired interaction between the hydroxyl group and amino group of chitosan. Hydroxyl ions that have higher mobility as compared to polyphosphate anions will be counteracted by the H^+ ^ions at acidic pH. Hence maximum probability for the interaction between polyphosphate anion and the amino group of chitosan (25) can be anticipated. Experimentally, the concentrations of chitosan and TPP that yielded opalescent suspensions were chosen for preparation of drug loaded nanoparticles and zone of formation was determined (Figure 2). The concentrations of chitosan and TPP were selected and different runs of experiment were conducted in accordance to 3^2^ full factorial design along with an extra design check point (N10). NFX loaded nanoparticles were obtained that were characterized. 

*Characterization of nanoparticles*

*Particle size and zeta potential*

The average particle size of NFX loaded chitosan nanoparticulate formulations (N1-N9) was found to be in range from 158.4 to 345.8 nm with PDI value ranging from 0.071-0.470 (Table 3). Formulation N_1_ containing least amount of chitosan and TPP had minimum particle size of 158.4 nm. Least levels of the reactants presented minimum concentration of protonated polymeric chain and the crosslinking anion to form crosslinked structure by the electrostatic interaction between the amino group of chitosan and polyphosphate anion (25). A gradual increase in particle size was observed with increased levels of either chitosan or TPP. Thus it can be concluded that the precise control of level reactants can ensure nanoparticles of desired size. Thus, for a given level of TPP, as the chitosan concentration was increased, the particle size increased. This may be reasoned to the entanglement of greater number of polymeric chains with polyphosphate anions (26). Same was true for varying the concentrations of TPP at constant chitosan concentration. Increasing TPP enabled more crosslinking leading to formation of larger particles. An invariably large particle size of 345.6 nm was observed for formulation N3 made with 0.1% w/v chitosan and TPP. This could be due to abundant availability of polyphosphate anion overriding the available polymeric chains, leading to higher crosslinking of nearby polymeric chains. Other reason could be availability of free polyphosphate anions on the surface of nanoparticles as well as in solvent system which might have resulted in aggregation of nanaoparticles (7). Optimum particle size was observed for formulation N4 when chitosan to TPP ratio was 3:1. 

The polydispersity index indicates the homodispersity of the nanoparticles; lower the PDI value monodisperse is the system (26). The PDI value of N1-N9 ranged from 0.071 to 0.470 (Table 3), and highest PDI value was recorded for formulation N3 (0.470) which might be due to aggregation of the nanoparticles. In N3, made with highest chitosan to TPP ratio, the free amino groups at surface of chitosan might have been masked by the free TPP ions resulting in insufficient electrostatic repulsive force that might have led to particle aggregation (27). Zeta potential largely affects the stability of nanoparticles through the electrostatic repulsion acting between the charged particles, generally particles with surface potential between -30 to +30 mV are considered to be stable system (16). Zeta potential of the formulations was in the range of +28 to +48 mV (Table 3). The positive surface potential of nanoparticles was attributed to the presence of free amino groups on the surface of nanoparticles, which is further linked to the chitosan concentration (17). All formulations exhibited zeta potential above +30 mV except formulation N3 exhibiting least zeta potential. This reduced surface potential can be ascribed to the fact that the amount of polyphosphate anion might have overridden the free amino group, hence generating a shielding effect on the charged nanocarriers and yielded decreased surface charge (9, 27). Thus the concentrations of chitosan and TPP not only govern the particle size of the formulated system but also the stability of the system. 

*Entrapment efficiency*

Entrapment efficiency of the formulations varied between 64.77- 77.38% (Table 3). Highest encapsulation efficiency was observed with intermediate level (0.15% w/v) of chitosan in comparison to the low and high level of chitosan. With the increase in polymer concentration from low to intermediate level at constant TPP concentration an increase in entrapment efficiency was observed. This increase in entrapment efficiency could be attributed to increased availability of polymeric chain to entrap the drug while on further increasing the polymer concentration decrease in encapsulation efficiency was recorded. The result witnessed could be ascribed to the fact that with increased concentration of polymer, polymeric chain interaction dominates drug polymer interaction and consequently less amount of drug gets entrapped in the polymeric matrix (17). The other reason for fall in entrapment efficiency with raised polymer concentration could be viscosity of the polymeric solution, as with increased polymer concentration viscosity of system increases which hinders the molecular movement of drug around polymeric chains and consequently reduced entrapment was observed (16). An increase in entrapment efficiency was observed with rising concentration of TPP at constant chitosan concentration. The enhancement of entrapment efficiency observed could be due to increased matrix formation at greater concentration of TPP. 

*In-vitro drug release*

**Figure 3:**
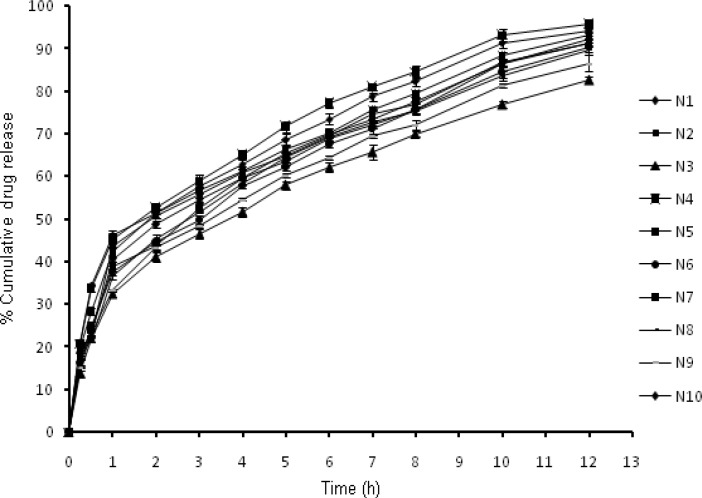
*In-vitro* release profiles of nanoparticles N1-N10.

The *in-vitro *release profiles (Figure 3) illustrates an initial burst release phase for 1 h followed by a sustained release phase for 12 h. Drug release during the burst release phase ranged from 32.34 to 46.35 % while the sustained release phase yielded a drug release from 82.61- 95.74% (Table 3). This initial fast release from the nanoparticles may be attributed to rapid hydration of hydrophilic matrix formed by chitosan leading to easy penetration of release media thus initiating dissolution of entrapped drug. Thus it could be proposed that the factor determining the initial drug release from nanoparticles is its solubilization or dissolution rate in release media (17). Further it is well known that NFX exhibits pH dependent solubility with highest solubility at acidic pH and least solubility at physiological pH (28). In this sense it is quite clear that the sink conditions were maintained during the study. Other factor apart from drug solubilization in release media governing the drug release includes extremely small size in nanometric range providing a greater surface area resulting in rapid release of the surface adsorbed drug (29). The results obtained clearly indicated that formulations N1 and N4 with smallest particle size show highest percentage of burst release. The effect of chitosan and TPP on drug release from the nanoparticles could be a result of other factors which are controlled by the amount of chitosan and TPP, such as particle size, crosslinking density and entrapment efficiency. The result for burst release phase show that with the increase in particle size from 158.4 to 345.8 nm the release rate decreased from 46.35 to 32.34%. The result obtained is in agreement of the fact that burst release is inversely related to the particle size as with increasing particle size, effective surface area decreases and consequently a significant decrease in burst release was witnessed. Similar effect was observed on cumulative drug release at 12^th ^h. Thus an indirect effect of chitosan and TPP concentration was observed on drug release from nanoparticles.

Yet another factor responsible for modulating drug release includes crosslinking density, which depends upon the ratio of chitosan and TPP used. Addition of TPP in higher concentration might have caused compact packaging and rigidity as well as increased inter chain bonding thereby decreasing drug release from nanoparticles. With raising chitosan concentration, viscosity of system increases leading to development of compact particles upon addition of TPP. As a consequence of higher crosslinking density system shows less swelling ability and decreased erosion rate and further a decrease in drug release rate was observed (16).

**Table 3:** Evaluation characteristics of norfloxacin nanoparticles

**Formulation code **	**Particle size ** **(nm) **	**PDI **	**Zeta potential (mV)**	**pH ** **(± SD) **	**% Entrapment efficiency ** **(±SD) **	**%CDR at 1h (±SD) **	**% CDR ** **at ** **12h ** **(± SD) **
N1	158.4	0.270	+38	6.04 ±0.02	64.77 ± 0.37	46.35±1.09	94.49 ± 0.69	
N2	192.8	0.324	+31	6.09± 0.01	68.27 ± 0.18	43.81±0.74	91.37 ± 0.50	
N3	345.8	0.470	+28	5.92 ±0.03	68.95 ± 0.49	32.34±0.60	82.61. ± 0.87	
N4	164.2	0.071	+32	5.85 ±0.01	74.51 ± 0.33	45.37±1.27	95.74 ± 0.92	
N5	215.6	0.120	+42	5.94± 0.03	76.42 ± .21	42.24±1.27	93.11 ± 0.36	
N6	243.8	0.181	+39	6.1 ± 0.02	77.38 ± 0.29	37.65±0.67	90.30 ± 0.43	
N7	256.1	0.210	+46	6.05± 0.02	70.17 ± 0.46	38.80±1.21	92.18 ± 0.19	
N8	298.4	0.180	+41	6.18 ±0.03	71.88 ± 0.87	36.75±0.89	89.63 ± 0.36	
N9	310.6	0.235	+39	6.21± 0.13	73.21 ± 0.26	33.31±0.40	86.54 ± 1.52	
N10*	164.6	0.112	+37	6.13 ±0.11	72.65 ±0.87	40.55±0.68	91.23 ± 1.48	

* Extra design check point

Kinetic modeling of the release profiles (Table 4) showed Higuchi model as the best fit for initial burst release phase of 1 h, suggesting Fickian diffusion type drug release pattern. While the sustained release phase till 12 h showed highest correlation for Korsmeyer Peppas model with the critical value of n>0.5. The results indicate an anamolous diffusion process i.e. combination of both diffusion of drug from the polymeric matrix and erosion of polymer matrix (30). This is further supported by the fact that the release of drug from nanoparticles is a sequential process, which includes three phases of drug release; desorption from surface, diffusion through swollen polysaccharide matrix and final step includes release due to erosion of polymeric matrix (17). As NFX is a small drug molecule hence it is assumed that the first two phases might have occurred simultaneously which includes surface desorption and diffusion of drug from the hydrated polymeric matrix yielding an instantaneous release on coming in contact with the release media followed by the third erosion based drug release phase (16, 30). 

**Table 4:** Kinetic modeling data of norfloxacin nanoparticulate formulations (N1-N9

**Formulation code**	**Burst release phase**				**Sustained release phase**				
	**Zero order**	**First order**		**Higuchi**	**Zero order**	**First order**	**Higuchi**	**Peppas**	
								R2	n
N1	0.911		0.638	0.994	0.981	0.981	0.965	0.984	0.589
N2	0.972		0.694	0.955	0.981	0.988	0.96	0.976	0.495
N3	0.939		0.674	0.994	0.983	0.948	0.978	0.994	0.565
N4	0.906		0.636	0.994	0.948	0.902	0.993	0.998	0.566
N5	0.883		0.632	0.986	0.965	0.945	0.968	0.988	0.550
N6	0.896		0.639	0.997	0.965	0.912	0.972	0.978	0.507
N7	0.934		0.65	0.989	0.955	0.894	0.980	0.982	0.511
N8	0.933		0.653	0.982	0.973	0.932	0.986	0.997	0.502
N9	0.949		0.675	0.977	0.982	0.947	0.974	0.991	0.521

*Statistical analysis*

The data obtained for the responses: % cumulative drug release (Y_1_), % entrapment efficiency (Y_2_) and particle size (Y_3_) were analyzed and fitted into various polynomial models for 3^2^ randomised full factorial design. It was observed that responses Y_1_, Y_2_ best fitted in quadratic response surface models while Y_3_ best fitted into quadratic response surface models on logarithmic transformation of the data. The polynomial equations showing relationship between the independent variable and responses Y_1_, Y_2_ and Y_3_ are expressed in following equations.

$Y_{1}=93.76-0.02X_{1}-3.83X_{2}+1.56X_{1}X_{2}-3.58X_{1}^{2}-1.06X_{2}^{2}$………Equation 2

$Y_{2}=76.56+2.21X_{1}+1.68X_{2}-0.29X_{1}X_{2}-6.56X_{1}^{2}-0.69X_{2}^{2}$ ……. Equation 3

$\ln Y_{3}=5.31+0.13X_{1}+0.23X_{2}-0.15X_{1}X_{2}+0.20X_{1}^{2}+0.21X_{2}^{2}$……. Equation 4

The above mentioned polynomial equations comprise of coefficients for intercept, first order main effect, interaction terms and quadratic terms. The negative sign in the equation indicates an antagonistic effect while positive sign indicates a synergistic effect (11). The response Y_1_ is antagonistically effected by the linear contribution of X_1_ and X_2_ and quadratic contribution of X_1_ and X_2_, while synergistically affected by the interaction terms X_1_X_2_. 

Similarly the response Y_2_ is antagonistically affected by interaction terms and quadratic contribution of X_1_ X_2_ while synergistically affected by linear contribution of X_1_ and X_2_. While the response Y_3_ (particle size) was synergistically effected by linear contribution of X_1_ X_2_ and quadratic contribution of X_1_^2^ and X_2_^2^ and antagonistically effected by the interaction term X_1_X_2_. The summary of polynomial equations with response effect and p-value are presented in Table 5A. 

The polynomial models were further analyzed by ANOVA to estimate significance of response models. The result revealed that the models were significant with R^2^> 0.9 (Table 5B) without significant lack of fit with a close coincidence between the adjusted and predicted R^2^ value. The coefficient of variance for all the responses were low indicating reliability of the experiment been carried out. Adequate precision which is indicator of signal to noise ratio was found to be adequate (>4) and determined the reliability of result obtained. On analysis of internally studentized residual versus predicted and experimental run, all data points were within limit (-3 to +3) indicating good fit of model with no outlying points. 

**Table 5:** Tabulated statistical evaluation of the responses and validation of experimental design

**A. Summary of each factor effect on responses Y**_1 ,_ **Y**_2 _ **and** **Y**_3_												
**FACTOR**	**Y**_1_				**Y**_2_				**Y**_3_			
	**Factor effect**		**P value**		**Factor effect**		**P value**		**Factor effect**			**P value**
X_1_	-0.020		0.0133		2.21		0.0013		0.13		0.0508	
X_2_	-38.3		0.9756		1.68		0.0016		0.23		0.0128	
X_1_X_2_	1.56		0.0034		-0.29		0.0036		-0.15		0.0673	
X_1_^2^	-.358		0.1064		-6.56		0.3303		0.20		0.0716	
X_2_^2^	-1.06		0.0281		-0.69		0.0003		0.21		0.7957	
**B.** Model summary statistics for quadratic response surface model												
**RESPONSE FACTOR**		**F-value**		**P>F**		**R** ^2^		**Adequate precision**		**C.V**		
Y1		13.65		0.0133		0.9317		11.406		1.66		
Y2		110.56		0.0013		0.9946		30.934		0.69		
Y3		10.80		0.0391		0.9474		8.786		1.92		
**C.** Validation of experimental design by evaluation of extra design check point formulation N10												
**Evaluation parameters **		**Predicted value**		**Actual value**		**% Error**		**Evaluation parameters **		**Predicted value**		
Particle size (nm)		167.03		164.6		1.823		Particle size (nm)		167.03		
% Entrapment efficiency		74.38		72.65		2.381		% Entrapment efficiency		74.38		
% CDR after 12 hrs		91.39		91.23		0.175		% CDR after 12 hrs		91.39		

**Figure 4:**
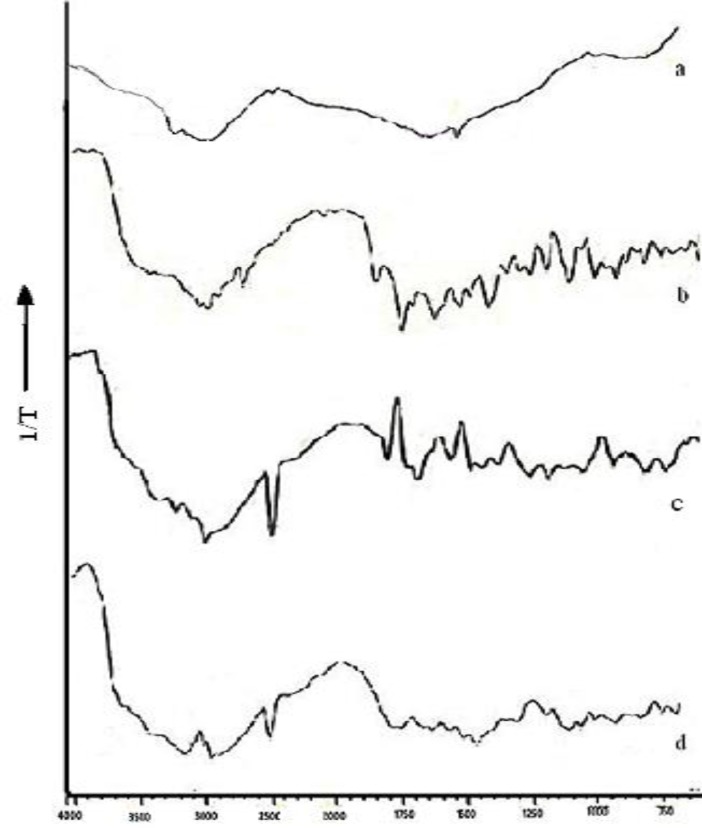
Diffuse reflectance spectra: (a) Nanoparticles N4, (b) Norfloxacin, (c) Chitosan, (d) Physical mixture

3-D response surface plots (Figure 4) were created by using the model generated. The response surface plot for Y_1_ (% CDR) displayed a curvilinear relationship with the varying concentration of chitosan. As evident from figure (4a) the region of intermediate chitosan concentrations at all concentration of TPP displayed maximum drug release. While when considering the effect of TPP an antagonistic linear relation was displayed. The response surface plots for % entrapment efficiency clearly depicts that intermediate concentration of chitosan yields maximum entrapment efficiency. As evident from the response surface plot that more pronounced effect on entrapment efficiency is generated on varying the chitosan concentration as compared to TPP concentration. On analyzing the response surface plot for particle size fig 4c a synergistic linear relationship between varying concentration of chitosan, TPP and particle size was observed. The plot clearly indicated that an increase in particle size was encountered when either of the independent variables concentration was varied. 

*Validation of design and optimization*

**Figure 5:**
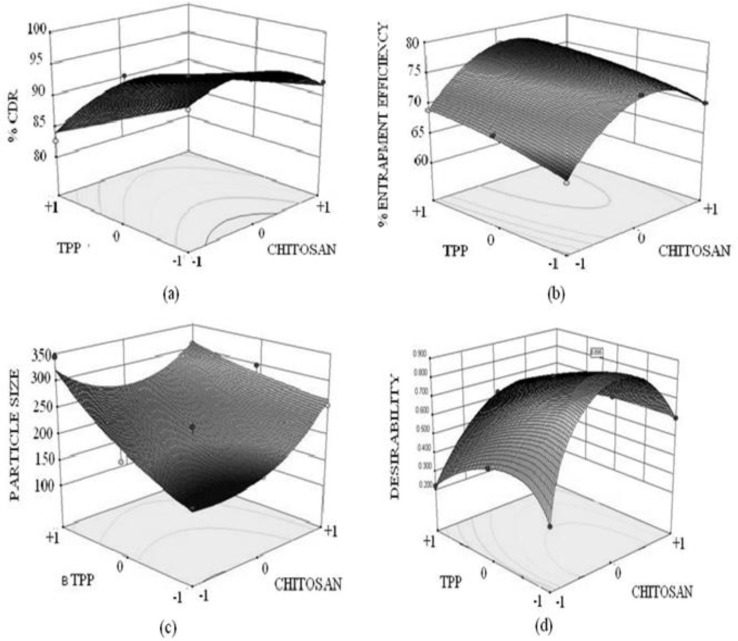
3 D Response surface plots showing combined effect of chitosan and TPP on (a) % cumulative drug release, (b) % entrapment efficiency, (c) Particle size, (d) Desirability of formulation

Validation of design was performed by construction of an extra design check point (N10) with lower intermediate level (-0.5) of chitosan and (-0.5) TPP. The % CDR, % entrapment efficiency, and particle size were found to be close with predicted values with low value of % Prediction error (Table 5C) which indicates reliability of developed mathematical model. A numerical optimization technique using the desirability approach was used to select NFX-loaded nanoparticulate formulation with desired response. Optimization was done with the aim to determine the optimum concentration of chitosan and TPP. Constraints applied to design were maximizing % cumulative drug release, % entrapment efficiency and minimizing particle size. Figure 5d portrays the change in desirability with changing concentration on chitosan and TPP. The optimal concentrations of chitosan and TPP for getting desired responses were 0.15% w/v and 0.05% w/v respectively (N4). Thus N4 was characterized by diffuse reflectance spectroscopy and later on developed as *in-situ* gels in carbopol base and a total of five formulations were screened.

*Diffuse reflectance spectroscopy*

DRS study helps to determine the physical or chemical interaction that may have taken place between the drug and polymeric matrix. The DRS spectra of free chitosan revealed the presence of peaks at 3300 cm^-1^ representing the free hydroxyl group overlapped with the –NH stretch at 3122 cm^-1 ^( Figure 5c). The characteristics peak of chitosan are the peak at 1575 cm^-1^due to presence of amino group, peak at 1320 due to N-acetyl glucosamine, C-H stretch at 2879- 52 cm^-1^, -CO group stretch at 1094 cm^-1^ while that for N-H bond at 1598-1600 cm^-1^. NFX exhibited two proton binding sites i.e. carboxyl and piperazinyl group with characteristic peaks at 1487 cm^-1^ for quinolone ring C-C and C-N stretch, 3326.98 cm^-1^ for N-H and O-H stretch, aromatic –CH stretch at 3043.46 cm^-1^, carboxylic acid C=O stretch at 1730 cm^-1^, and C=C stretch at 1619 cm^-1^ ( Figure 5b). When norfloxacin was entrapped in the chitosan nanoparticles only slight shift in stretch peak of –OH to 3315 cm^-1^ (Figure 5a ) was observed while the peak of quinolone ring was observed at 1487 cm^-1^ indicating no interaction between the entrapped drug and polymer. This can also be justified by the fact that norfloxacin exists in cationic form at acidic pH (pH of reaction medium) hence there is least possibility of electrostatic interaction between chitosan and NFX (28). While a shift in peak of amino group of chitosan from 1575 cm^-1^ to 1598 cm^-1^ was observed indicating formation of new amide bond as a result of ionic interaction between chitosan and TPP. Figure 5d is additive spectra of the physical mixture of chitosan and NFX used as reference. 

*Evaluation of nanoparticulate in-situ gel of N4*

*Clarity and optical transmittance*

For ophthalmic delivery clarity is of prime importance. Visually the formulations N4G1-N4G5 were clear and the clarity was confirmed by optical transmittance measurements. All the formulations showed optimum optical transmittance > 90% and the highest optical transmittance was recorded for N4G5 (96.33% ± 0.57). Though made with highest concentration of carbopol but in the gel state optical transmittance depends upon the crosslinking density which increases when concentration of carbopol increases (19). 

*pH and drug content*

The pH of an ocular formulation is one of the pivotal measures of its tolerability and in present case it is a critical determinant of gelling ability. Both aspects are essential components of patient compliance. The pH of the *in-situ* gels was in the range of 5.84 to 6.25 (Table 6) that can be tolerated by human eye. The pH tolerability of ocular tissues is reported to be 5-9 (35). The drug content closely varied from 96.88 to 99.24% indicating homogeneity of the formulations.

**Table 6:** Evaluation characteristics of nanoparticulate *in-situ* gels of norfloxacin

**Gel** **code**	**Clarity**	**Optical transmittance (%)**	**pH**	**Drug content**	**Gelling capacity**
N4G1	Transparent	91.88 ± 0.80	6.25 ± 0.86	99.24 ± 2.38	-
N4G2	Transparent	92.20 ± 0.72	6.17 ± 0.41	96.88 ± 1.63	+
N4G3	Transparent	94.36 ± 0.66	6.02 ± 0.53	98.74 ± 1.18	++
N4G4	Transparent	95.66 ± 0.57	5.96 ± 0.58	97.38 ± 2.35	++
N4G5	Transparent	96.33 ± 0.57	5.84 ± 0.63	98.31 ± 1.39	+++

*In-vitro gelling*

Gelling ability infers the speed and extent of gelation and is measured as the time taken for gel formation and gel stability. While formulating a pH transforming gel the sol should have an optimum viscosity to allow easy instillation and subsequently undergo transformation into gel on application. The sol-gel transformation should yield formulation of optimum gelling ability (34, 20). Considering, the ability of aqueous solution of carbopol 934 P, to transform into a stiff gel with rising pH, different concentrations of carbopol (0.1-0.5% w/v) were investigated for their gelling ability and graded as shown in Table 6. With increase in carbopol concentration an increase in gelling ability was observed and N4G5 formulation with +++ sign was assigned strongest gelling ability and N4G1 formulation − sign with was assigned no gelling ability. The gelling phenomenon can be explained by increased ionization of functional groups present in carbopol 934P as a result of increasing pH, leading to an increased electrostatic repulsion between adjacent –COOH groups and the subsequent expansion of polymeric network (33, 20). Furthermore, formation of stiffer gel might be due to hydrophobic nature of carbopol 934P backbone leading to development of hydrophobic interchain aggregation (34). Thus higher the carbopol level more gelling ability is expected. Consequently, N4G5 with highest carbopol 934P concentration of 0.5% w/v showed best gelation ability. *In-situ* gels made with lower concentrations of carbopol (N4G1 to N4G4) did not exhibit desired gelling characteristics and hence were abandoned. Thus based on physicochemical attributes N4G5 was persuaded for further assessment.

*Viscosity*

**Figure 6:**
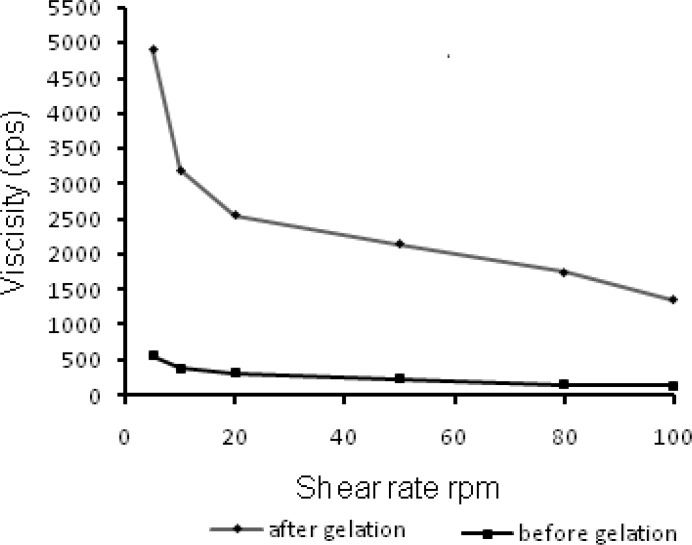
3D Response surface plots showing combined effect of chitosan and TPP on (a) % cumulative drug release, (b) % entrapment efficiency, (c) Particle size, (d) Desirability of formulation

The residence time of the ophthalmic formulation at the precorneal surface is affected by its viscosity that is of importance for enhancing the residence time. In context to ocular physiology, the range of shear rate experienced during relative movement of eyelids and globe is extremely wide ranging from 0.03 to 0.14 s^-1^ during inter blinking period to 4250-28500 s^-1^ during a blink. Thus the viscosity of formulation should not be such that it disturbs the pseudoplastic behavior of tear film in the eye (31). Hence it is advisable to use a polymer with pseudoplastic character (20). Such polymeric formulations have low viscosity at high shear rate and higher viscosity at low shear rate therefore expected that such formulation would not hinder the reflex blinking and consequently provide better patient compliance. The rheological profile of N4G5 (Figure 6) before gelation, displayed insignificant changes in viscosity on increasing shear rate. However, the profile of gelled formulation depicted a clear transition of viscosity decrease with increased shear rate which could be ascribed to the pseudoplastic character of the *in-situ* gelling formulation. Thus the formulation can be efficiently used for ophthalmic administration. 

*In-vitro release*

**Figure 7:**
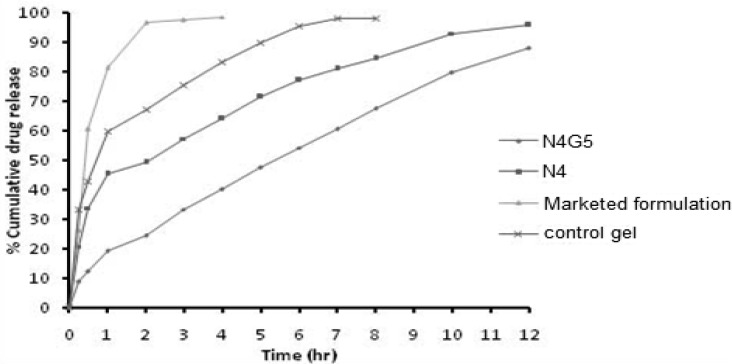
Comparative ex vivo transcorneal permeation profiles of nanoparticulate in-situ gel (N4G5) and marketed formulation

The cumulative drug release from nanoparticulate *in-situ* gel N4G5 at 12 h was found to be 88.01 ± 0.48 % (Figure 7). The release profile when fitted to kinetic models exhibited highest r^2^ for Higuchi model (0.994). Overall curve fitting of data revealed sustained release behavior with diffusion as the major mechanism of drug release. The proposed release mechanism from the nanoparticulate *in-situ* gel includes first the random coiling of polymeric network in response to pH changes and thereafter, on gelation of polymeric chains to form the matrix system laden with nanoparticles gel, the drug was released by diffusion through the resultant gel matrix (34). The release profile of N4G5 was compared to optimized nanoparticulate formulation N4, marketed formulation and control gel. A significant difference was found between the release profiles at 95% confidence interval. Similarity factor was determined for the different formulation as compared to the marketed formulation (F2>50) which show a considerable difference between the release profiles when compared to marketed formulation. The release profile of N4G5 advocates its greater sustaining ability as compared to the control gel and marketed formulation. Drug release from N4G5 was to a lower extent than N4 that can be described by Stokes Einstein equation according to which viscosity influences the diffusion of drug inversely (21). 

*Transmission electron microscopy*

**Figure 8:**
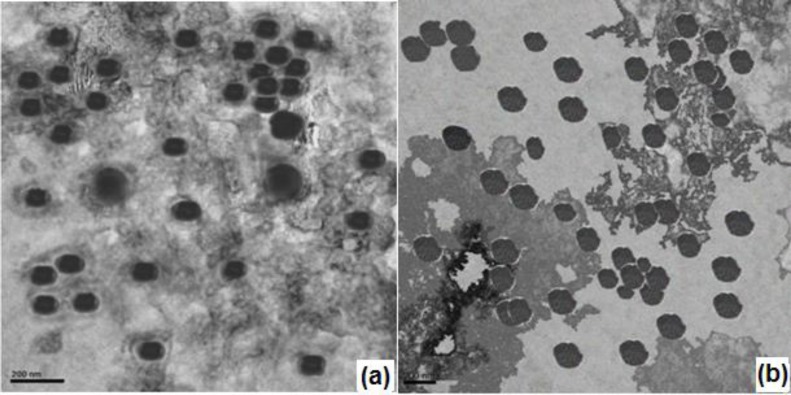
TEM images (a) Optimized nanoparticulate in-situ gel (N4G5), (b) Optimized nanoparticles N4.

The TEM image of nanoparticulate *in-situ* gel N4G5 (Figure 8a) showed spherical structure comparable to optimized nanoparticulate formulation N4 (8b). The size of nanoparticles was in conformity with the results obtained for zeta sizing (Table 3). The images for nanoparticulate enmeshed in the *in-situ *gel matrix displayed a mild loss in spherical boundaries probably due to the gel matrix stress on the nanoparticles. 

*Ocular mucoadhesion strength*

Mucoadhesive strength of nanoparticulate N4G5 was found to be 1137.45 dynes/cm^2^ which is approximately 8 times higher than the ocular shear force of 150 dynes/cm^2^ (33). The result obtained thus signifies that there a significant mucoadhesive force will exist to resist the shear during the reflex blinking. Thus the formulation would be able to achieve desirable residence in the precorneal area.

*Corneal toxicity*

**Figure 9:**
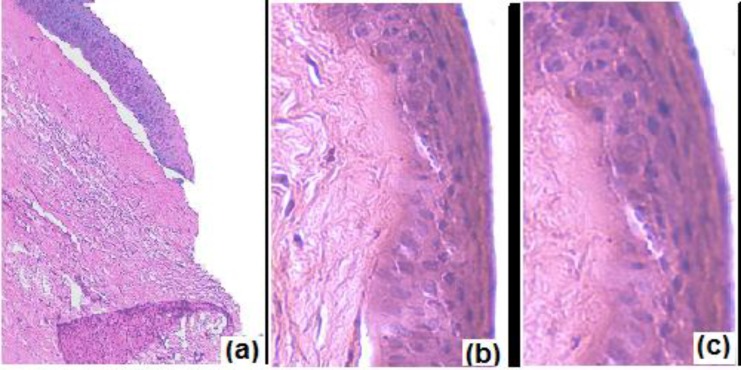
Histological cross section of excised goat cornea, stained with haematoxylin-eosin after incubation in (a) Normal saline (control); (b) N4G5; (c) isopropyl alcohol (positive control

The excised cornea incubated with isopropyl alcohol (positive control) was marked by widening of intracellular spaces. Deformation of cells was clearly visible with distortion of superficial epithelial cells (Figure 9a). The cornea incubated with normal saline (negative control; Figure 9b) showed no evidence of tissue damage. Similar findings were observed for cornea incubated with N4G5 (Figure. 9c) where the formulation did not display any destructive effect on corneal epithelium and stroma suggesting low corneal toxicity.

*Antibacterial activity*

**Figure 10:**
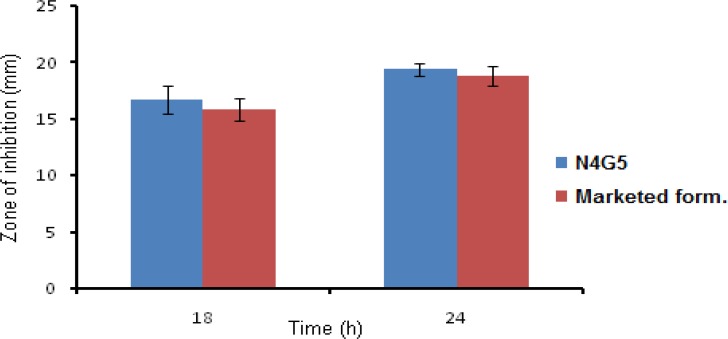
Bar chart showing zone of inhibition for nanoparticulate in-situ gel (N4G5) and marketed formulation

The test formulation (N4G5) showed clear zone of inhibition having a diameter of 16.71 ± 1.20 and 19.44 ± 0.54 cm at 18 and 24 h, respectively. The marketed formulation revealed zone of inhibition diameter of 15.84 ± 0.94 and 18.79 ± 0.88 cm at 18 and 24 h, respectively (Figure 10). Results revealed prolonged antimicrobial efficacy of developed nanoparticles compared to marketed formulation. On applying student t-test at 95% confidence interval no significant difference (P<0.5) was found between the antibacterial activity of N4G5 and marketed formulation.

*Ocular irritation test*

**Figure 11:**
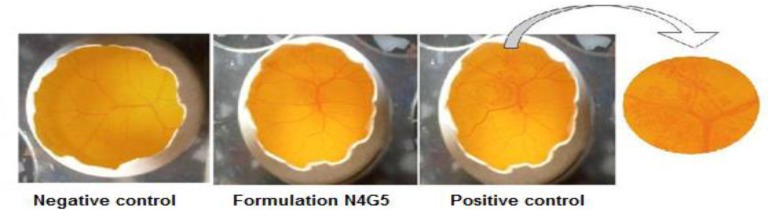
Images of HET-CAM test of N4G5 in-situ nanoparticulate gel of norfloxacin conducted on fertile eggs.

HET CAM (Table 7) revealed a mean score of zero for saline throughout the duration of study for 12 h. The CAM when exposed to N4G5 showed no signs of tissue inflammation at the end of 10 h (Figure 11) while a slight visible membrane discoloration in one test egg was observed at 12 h of study. Thus a mean score of 0.33 at 12 h was adjured to the formulation. Thus N4G5 can be designated as very slightly irritant. This might be due to presence of carbopol 934P in the N4G5 which shifted the pH of formulation towards acidic pH. The proposed remedy is adjustment of pH without compromising the performance of drug at its stability. The test formulation was compared with positive and negative controls. The scores obtained for positive and negative control were 3 and 0 respectively, which indicates the maximal ocular irritation caused due to positive control while no such sign of haemorrhage was evident from the negative control.

**Table 7:** Scoring of HET-CAM test of N4G5 in-situ nanoparticulate gel of norfloxacin conducted on fertile eggs

**Preparation**	**Egg **	**SCORE**						
		**Time (min)**						
		**0.5 **	**2 **	**5 **	**60 **	**240 **	**480 **	**720 **
0.9% NaCl (-ve control)	Egg 1	0	0	0	0	0	0	0
	Egg 2	0	0	0	0	0	0	0
	Egg 3	0	0	0	0	0	0	0
	Mean	0	0	0	0	0	0	0
Optimized formulations (N4G5)	Egg 1	0	0	0	0	0	0	0
	Egg 2	0	0	0	0	0	0	1
	Egg 3	0	0	0	0	0	0	0
	Mean	0	0	0	0	0	0	0.33
0.1 M NaOH	Egg 1	1	3	3	3	3	3	3
	Egg 2	1	3	3	3	3	3	3
	Egg 3	3	3	3	3	3	3	3
	Mean	1.88	3	3	3	3	3	3

0= Non irritant (No visible haemorrhage); 1= Mild irritant (Just visible membrane discoloration); 2= moderately irritant (Structures are covered partially due to membrane discolouration or haemorrhage); 3= severely irritant(Structures are covered totally due to membrane discoloration or haemorrhage).

## Conclusion

The developed chitosan nanoparticulate in-situ gel can be considered as an effective and superior dosage form over the commercially available eye drops. The developed system could be considered as an effective dosage form for treatment and eradication of extra ocular bacterial infection without compromising patient safety and compliance. Furthermore the presence of pH triggered in-situ gelling polymer carbopol lead to longer residence time of drug loaded nanoparticles due to its mucoadhesive character and increased erosion time of gel by the tear fluid. 
